# Real-time monitoring of the reaction between aniline and acetonylacetone using extractive electorspray ionization tandem mass spectrometry

**DOI:** 10.1038/s41598-019-55921-1

**Published:** 2019-12-17

**Authors:** Xinglei Zhang, Miaorong Pei, Debo Wu, Shuiping Yang, Zhanggao Le

**Affiliations:** Jiangxi Key Laboratory for Mass Spectrometry and Instrumentation, East China University of Technology, Nanchang, 330013 People’s Republic of China

**Keywords:** Mass spectrometry, Reaction mechanisms

## Abstract

In this work an on-line monitoring method was developed to study the mechanism of acetic acid catalyzed reaction between aniline and acetonylacetone using extractive electorspray ionization-tandem mass spectrometry (EESI-MS). The signals of reactants, intermediates and various byproducts were continuously detected as a function of reaction time. The chemical assignment of each signal was done via multi-stage collision induced dissociation (CID) analysis, and the reaction mechanism between aniline and acetonylacetone was deduced based on the generated molecular ions and fragment ions. The results indicate that on-line EESI-MS is an effective technique for the real time analysis of chemical reactions. EESI avoids off-line sample pretreatment and provides “soft” ionization, which allows direct analysis of various analytes at molecular level.

## Introduction

Pyrrole and its derivatives are a class of important five-membered nitrogen heterocyclic compounds, which can be synthesized by condensation from 1,4-dicarbonyl compounds, known as Paal-Knorr reaction. Though a variety of methods have been developed for pyrrole synthesis, Paal-Knorr condensation still remains the most widely used approach^1,2^. As is well known, the intermediates of an organic reaction may vary significantly depending on reaction conditions^3^. Therefore, an on-line analytical method is urgently needed to study the reaction pathway and mechanism. Basically, the chemical reaction mechanism is studied based on theoretical assumption combined with experimental validation^4–7^. Normally, in order to detect the intermediates and various byproducts, continuous sampling at different time intervals is needed during reaction. Therefore, certain reagents are added to quench any further reaction. In addition, before analysis of these samples using conventional techniques, such as high-performance liquid chromatography-mass spectrometry (HPLC-MS)^8^ or gas chromatography-mass spectrometry (GC-MS), the samples need to be preserved by cooling or adding more reagents. Consequently, the analytes in each step may suffer from loss or transformation of chemical species, thus greatly challenging further investigation^9–15^.

Extractive electrospray ionization-mass spectrometry (EESI-MS) is characterized by its high sensitivity, fast analysis and avoidance of sample pretreatment^8,16–22^. Therefore, EESI-MS is suitable for the real-time monitoring of chemical reaction process, enabling direct detection of various intermediates and byproducts during the entire reaction process^23,24^. The key part for on-line EESI-MS direct analysis of chemical reaction is the ion source. Conventional electron ionization (EI) and chemical ionization (CI) sources can only be used for gaseous molecules under vacuum conditions^18^. Electrospray ionization (ESI) and atmospheric-pressure chemical ionization (APCI)^25^ also can not be routinely used for the direct analysis of analytes with complex matrices. The EESI, however, is advantageous over these techniques, because it uses two separate sprayers: one to nebulize the sample solution and the other to produce charged microdroplets of solvent. This approach is based on liquid–liquid extraction between the colliding microdroplets, thus allowing direct sensitive detection of analytes without interference from complex matrices^26–33^.

Based on the extractive electorspray ionization mass spectrometry (EESI-MS), here we propose a mass spectrometry method for the real-time monitoring of reaction process between aniline and acetonylacetone catalyzed by acetic acid. The experimental conditions like ESI voltage, gas flow and position of glass tube, were optimized for sensitive detection of various intermediates and byproducts. By observing the behaviors of these components as a function of reaction time, the reaction process and mechanism were determined.

## Materials and Methods

### Reagents and instruments

Aniline (99%), acetonylacetone (99%), acetic acid (99%) and methanol (99%) were purchased from Aladdin Reagent (Shanghai, China). N_2_ (>99.99%, Guoteng Gas, Jiangxi, China) was used as carrier gas. All solutions were prepared with double-deionized (DDI) water (18.2 MΩ·cm).

**Figure 1:**
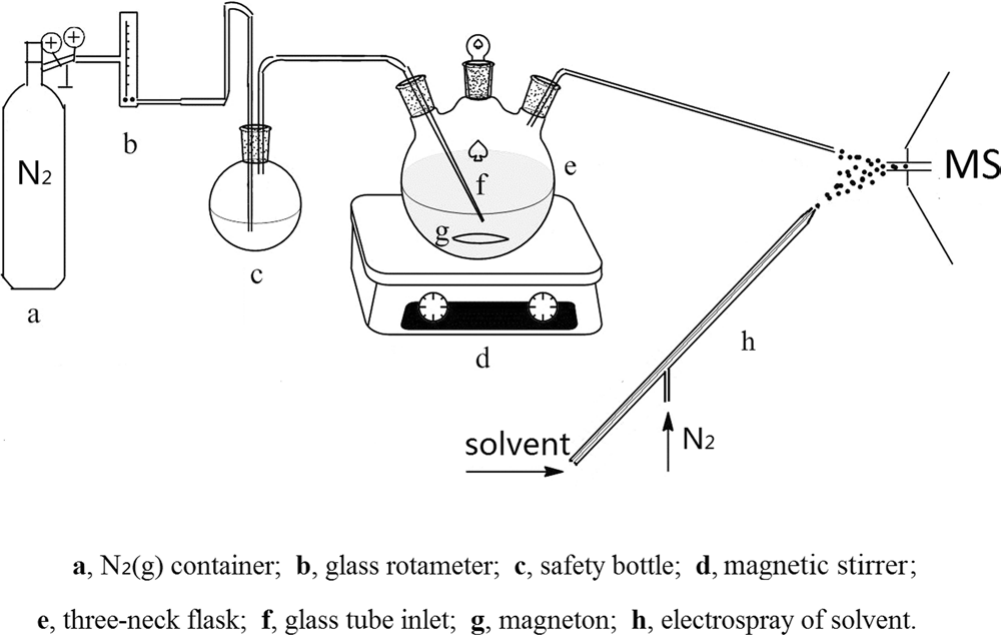
Schematic of on-line monitoring of acetic acid-catalyzed reaction between aniline and acetonylacetone using EESI-MS.

Previously reported electrospray extraction ionization source (EESI)^26,28,29^ was modified for on-line monitoring of chemical reaction (Fig. 1). Briefly, The produced intermediates during reaction were carried and sprayed out of a capillary by N_2_(g). The other sprayer was used to generate charged microdroplets of spray solvent (methanol). The analytes were then extracted by solvent microdropletes for further MS analysis. The angle between the two sprayers of EESI was about 38°, and the tip of the sprayers was about 5 mm away from the inlet capillary of mass spectrometer. LTQ-XL linear ion trap spectrometer (Finnigan, San Jose, CA**)** was used as detector for all the experiments. The analytes were detected in positive ion mode. The ion transport tube’s temperature was 150 °C. Mass range of detection was set from 50 to 500. Other parameters were set by default of LTQ-MS.

### Real-time chemical sampling

The setup of the real-time chemical sampling is shown in Fig. 1. A 25 mL three-neck round bottom flask was used as reactor, which was placed on a magnetic stirrer (Heze Xinyuan Instrument co., LTD.). A gas flowmeter (0.15–1.5 L/min) (Changzhou Edkorx Instrument co., LTD.) and a glass rotameter (LZB-3WB) were used to adjust the flow of carrier gas (N_2_) (0.3 MPa, 0.8 L/min). The gas pressure for electrospray was 1.0 MPa, also with N_2_ as carrier gas. Generally, N_2_ was introduced into the reaction vessel through glass tube. A 50 mL flask was used as safety bottle to buffer air flow and prevent solution from backflow. A peek tube was connected to the right neck of the reaction flask in order to transport analytes to mass spectrometer. The N_2_ flow rate was adjusted to 0.8 L/min by rotameter. N_2_ was then introduced into the reactor through glass tube, resulting in generation of continuous bubbles which then rose and carried various analytes up to the surface of solution. A micro spray was then formed by the fine droplets resulted from bubble bursting^34,35^. The chemical composition of formed micro spray therefore reflected the composition of the bulk solution. The formed micro spray then interacted with the charged droplets of electrospray solvent (methanol). As a result, the analytes in neutral aerosol were extracted and ionized efficiently for real-time detection by MS.

### Experimental procedure

First, the on-line detection device for the reaction between aniline and acetonylacetone was constructed as shown in Fig. 1. Then, the EESI and MS parameters (*e*.*g*. angle between two sprayers, flow rate of carrier gas, ESI voltage) were optimized to obtain the optimum operational conditions. In order to obtain stable signal stability, the position of gas inlet in the reactor (*e*.*g*. immersed in aqueous solution and stayed in the head space of the reactor) was investigated. In addition, the MS spectra of each reactant were also analyzed by tandem collision induced dissociation (CID) analysis. Finally, the reaction pathway and mechanism were studied in detail with 0.2 mmol aniline, 0.2 mmol acetonylacetone, 0.02 mmol acetic acid and 3 mL solvent of methanol added into the reactor.

## Results and Discussion

### Optimization of ESI voltage

**Figure 2:**
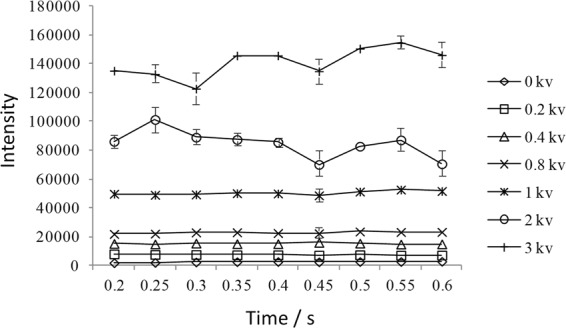
Influence of ESI voltage on signal intensity and stability of methanol.

The influence of ESI voltage on signal intensity and stability were investigated in the voltage range of 0–3 kV using methanol as analyte. It can be seen from Fig. 2 that with the increase of ESI voltage, signal intensity for methanol increased notably. However, the signal stability deteriorated obviously at the same time as voltage increased, which would have a great influence on on-line monitoring of the reaction. At low voltages (0–0.8 kV), though signal intensity was relatively stable, it was too weak to be detected. Consequently, a voltage of 1 kV was chosen as the optimum condition.

### Formation of stable microspray of analytes

**Figure 3:**
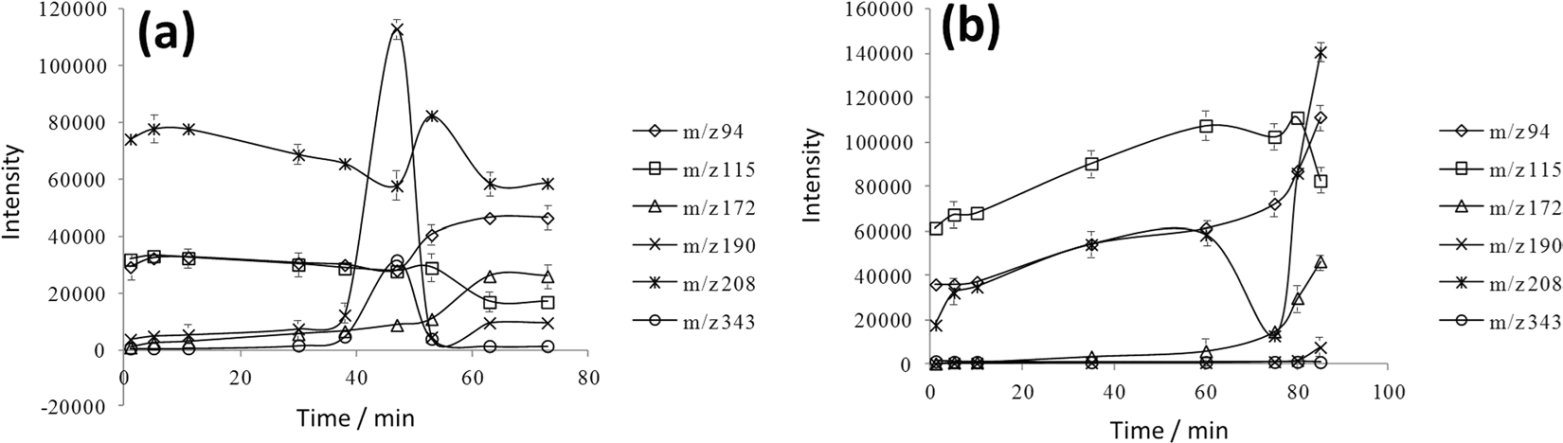
Signal intensity and stability of various analytes as a function of reaction time. (**a**) with the glass tube immersed in the solution; (**b**) with the glass tube located in the head space of reactor.

With 0.2 mmol reactant of aniline and acetonylacetone in the reactor, the reaction was then initiated by addition of 0.02 mmol acetic acid as catalyst. The experimental parameters of MS and EESI were adjusted as mentioned above. In order to generate stable micro spray of analytes for direct EESI-MS analysis, reference experiments were conducted with the glass tube immersed in the liquid phase and stayed in the head space of the reactor, respectively. It can be seen from Fig. 3(a) that when the glass tube was inserted in solution stable micro spay was formed, as indicated by the generally stable signal intensity of various analytes as a function of reaction time. The “peak” shape or gradual decrease (or increase) of each line was ascribed to the concentration changes of various species inside the reaction system. In contrast, unstable response signal were observed when the glass tube was located in the head space of the reactor, though the intensity for some analytes was even higher. In addition, a severe lag of signal response was observed as a function of reaction time (Fig. 3(b)). This was probably due to the fact that the concentration of the steam components in the head space of the reactor was dependent on their respective saturation vapor pressure. However, during the reaction the saturated vapor pressure of each component in the reactor may vary significantly. As a result, the equilibrium between vapor and liquid phase would be greatly affected and the formed micro spray could not reflect the components of the bulk solution accurately. Therefore, the first approach was found more suitable for the online EESI-MS analysis of chemical reaction.

### Mass spectra of reactants

**Figure 4:**
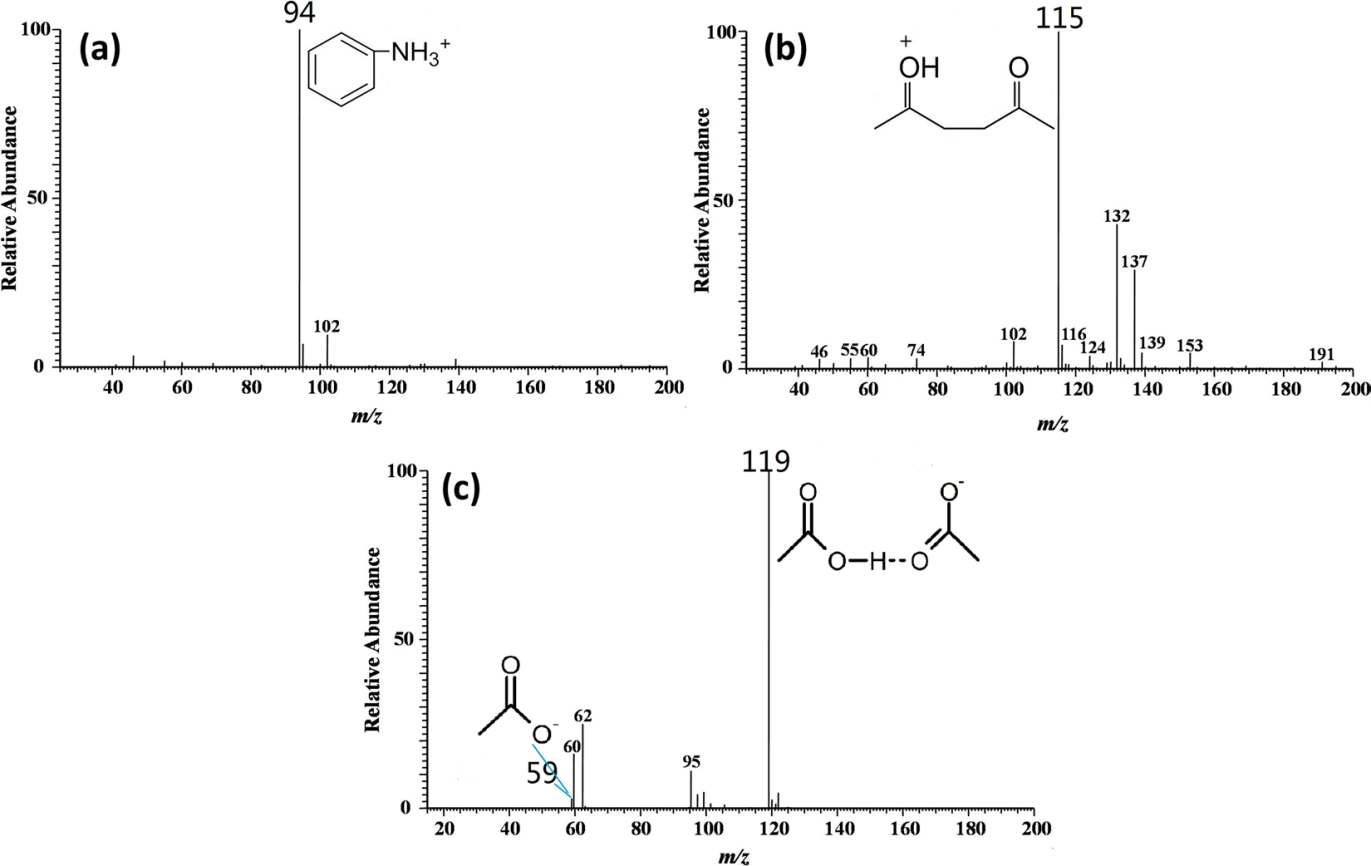
MS spectra of reactants and catalyst: (**a**) aniline; (**b**) acetonylacetone; (**c**) acetic acid.

In order to obtain the spectra information of the reactants and catalyst, individual aniline, acetonylacetone and acetic acid was analyzed under the same condition and multi-stage CID analysis was conducted. 3 ml of aniline, acetonylacetone and acetic acid was added into the flask separately. After introduction of N_2_, each analyte was nebulized and brought to mass spectrometer, and thus the spectra were recorded. As is shown in Fig. 4, aniline generated a peak at m/z 94 in the form of protonated [M + H]^+^. Acetonylacetone yielded three different forms of ions: acetonylacetone molecular ion at m/z 115, radical of acetonylacetone combined with water [M + H_2_O]^+^ · at m/z 132 and acetonylacetone sodiated [M + Na]^+^ ion at m/z 137 (Figs. 4 and S1). As for acetic acid, only one ion at m/z 119 was detected, which was the dimer of acetic acid. This could be explained by the fact that the hydroxyl hydrogen in acetic acid was able to form the intermolecular hydrogen bond easily and the charge distribution in molecular would be more concentrated during EESI process.

**Figure 5:**
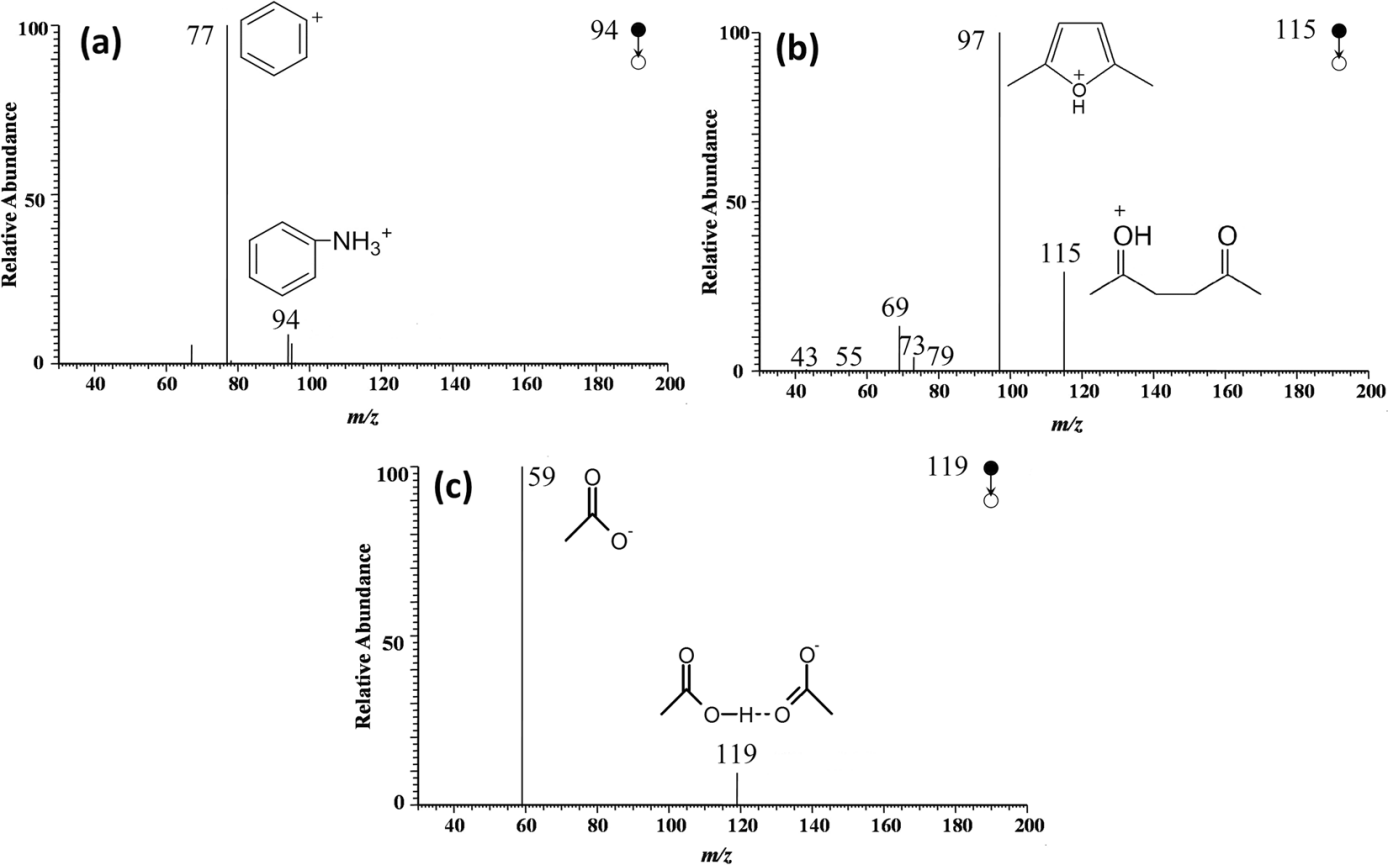
MS^2^ spectra of aniline (**a**), acetonylacetone (**b**) and acetic acid (**c**); and the possible fragmentation pathway.

Figure 5 presents the CID analysis for each signal mentioned above. Structurally, the six-member ring of aniline was stable and could not be broken easily. This was confirmed with CID analysis of the peak at m/z 94. With the elevation of collision energy, aniline (m/z 94) underwent the neutral loss of ammonia molecule (NH_3_) to generate positively charged benzene ring with the m/z value of 77. The acetonylacetone molecular ion at m/z 115 was able to lose H_2_O (mass of 18) to generate positively charged enol structure (m/z 97). The high intensity of m/z 97 signal in Fig. 5b suggested the formation of a stable structure, *i*.*e*. furan. Consequently, m/z 97 was concluded to be protonated 2,5-dimethylfuran, and the proposed mechanism of its formation was demonstrated in Fig. S2 in supporting information. With elevated energy, acetonylacetone molecular ion would yield positive ion of ethenone (m/z 43) due to the breaking of C-C bond. As for acetic acid, the intermolecular hydrogen bond was ruptured in CID mode and the dimer was broken into monomer (m/z 59).

### On-line real time monitoring of reaction

**Figure 6:**
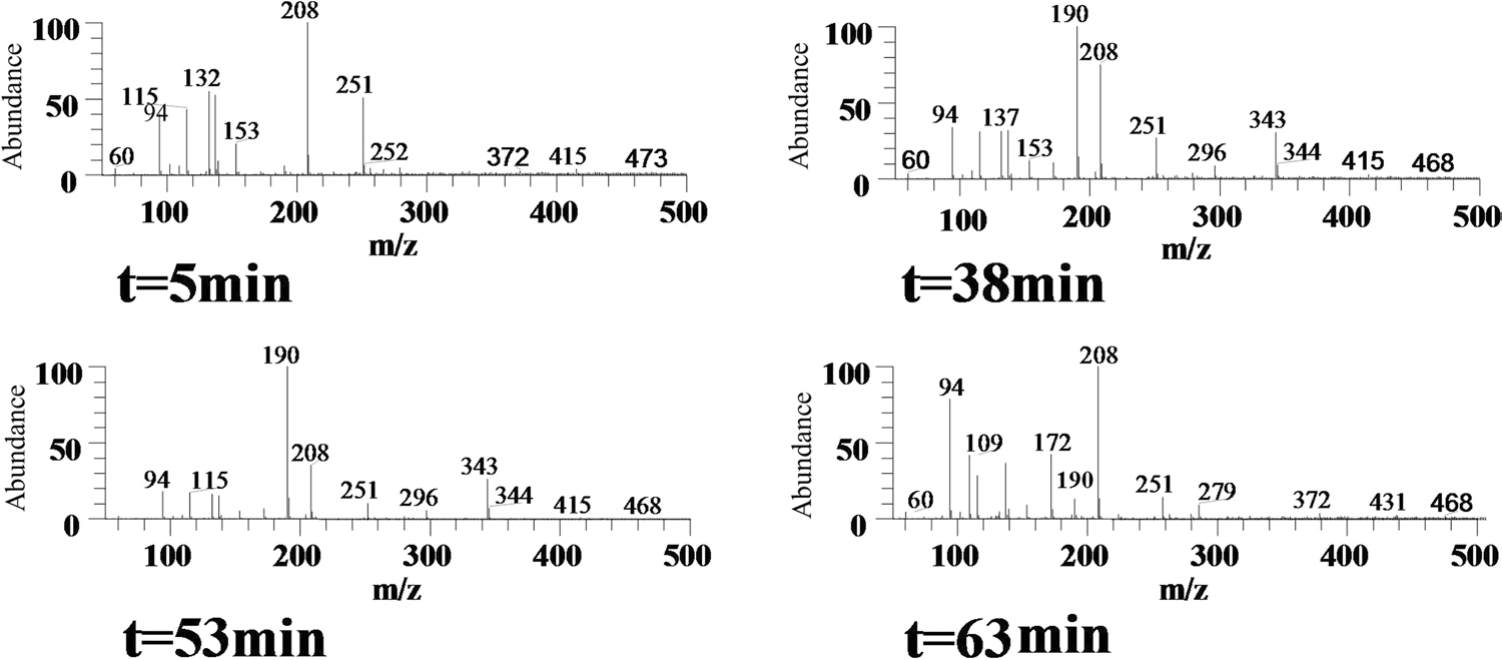
The MS spectra captured during the reaction between aniline and acetonylacetone using EESI-MS.

Figure 6 shows the spectra obtained at different time intervals during the reaction between aniline and acetonylacetone using the established method. Before the addition of acetic acid, only peaks of aniline (m/z 94) and acetonylacetone (m/z 115, m/z 132, m/z 251) could be detected. With addition of acetic acid as catalyst, the peak at m/z 208 was observed, which reached a relatively high intensity level at t = 5 min. With the increase of reaction time from 5 to 53 min, the intensity of reactants declined gradually. Meanwhile two new signals emerged: m/z of 190 and m/z 343. The ions of m/z 190 and m/z 343 significantly decreased or totally disappeared (t = 63 min) with the increase of reaction time. At t = 63 min, another signal at m/z 172 was detected. The structure of m/z 208, m/z 190 and m/z 343 were further confirmed by collision induced dissociation (CID) analysis.

It is worth noting that other adducts such as m/z 137, m/z 251, m/z 153, m/z 266 were also observed at substantial amounts in the spectra. They were produced due to the reaction between acetonylacetone and Na^+^, K^+^ ions, as illustrated in Fig. S1. This reaction occurred because the active α hydrogen in acetonylacetone was prone to dissociation, and these m/z signals correspond to the addition-product of Na^+^ or K^+^ to the molecule. As the reaction was carried further, both the acetonylacetone molecular and those combined with metal ions would participate in the reaction. Therefore, it did not affect the total amount of reactants. The intensity of sodiated [M + Na]^+^ and potassiated [K + Na]^+^ acetonylacetone ions decreased gradually with time until the reaction ended (Fig. S3).

### Qualitative analysis of intermediates

**Figure 7:**
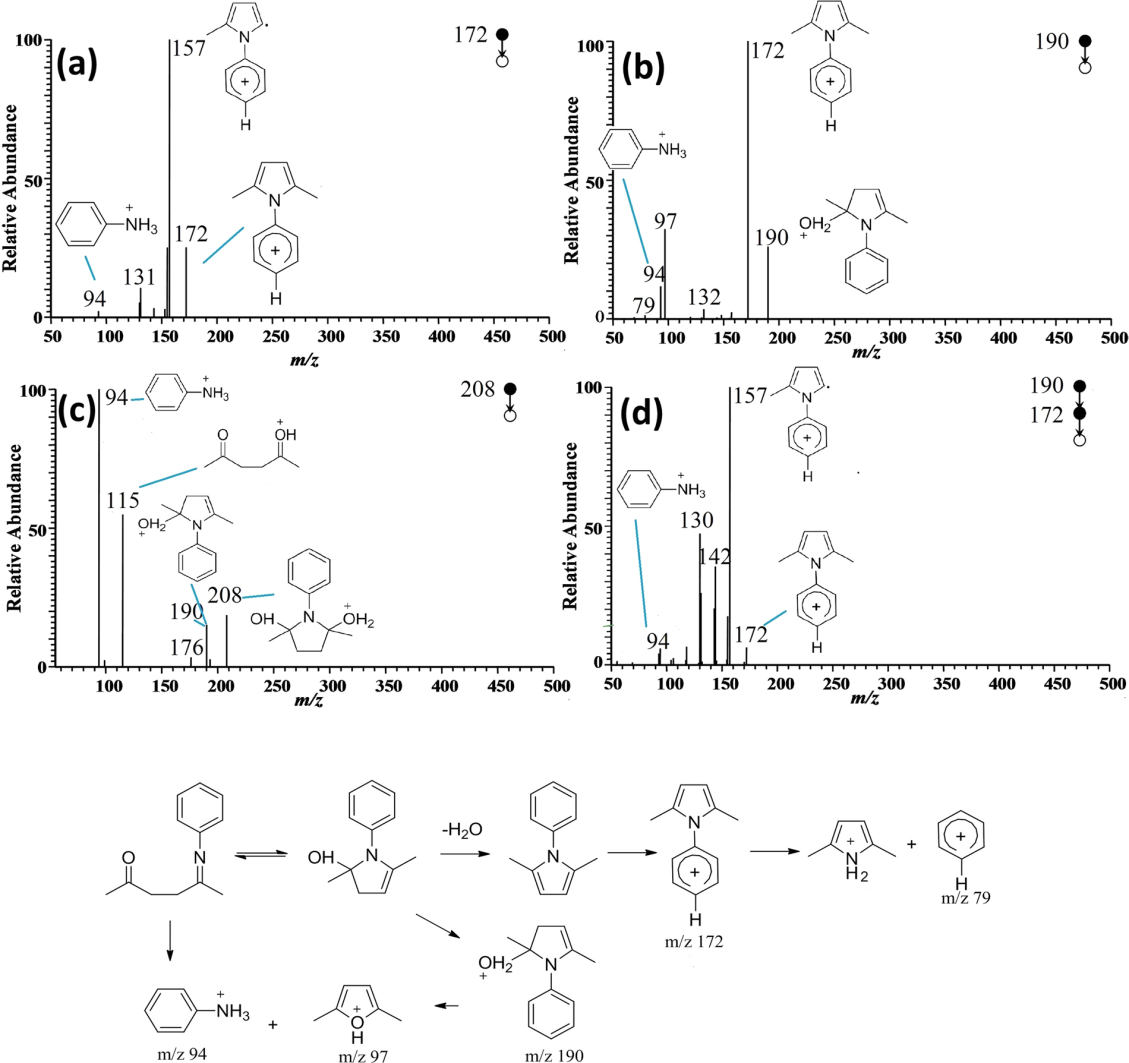
Spectra analysis of intermediates and possible fragmentation pathway. (**a**) MS^2^ spectrum of m/z 172; (**b**) MS^2^ spectrum of m/z 190; (**c)** MS^2^ spectrum of m/z 208; (**d**) MS^3^ spectrum of m/z 172.

**Figure 8:**
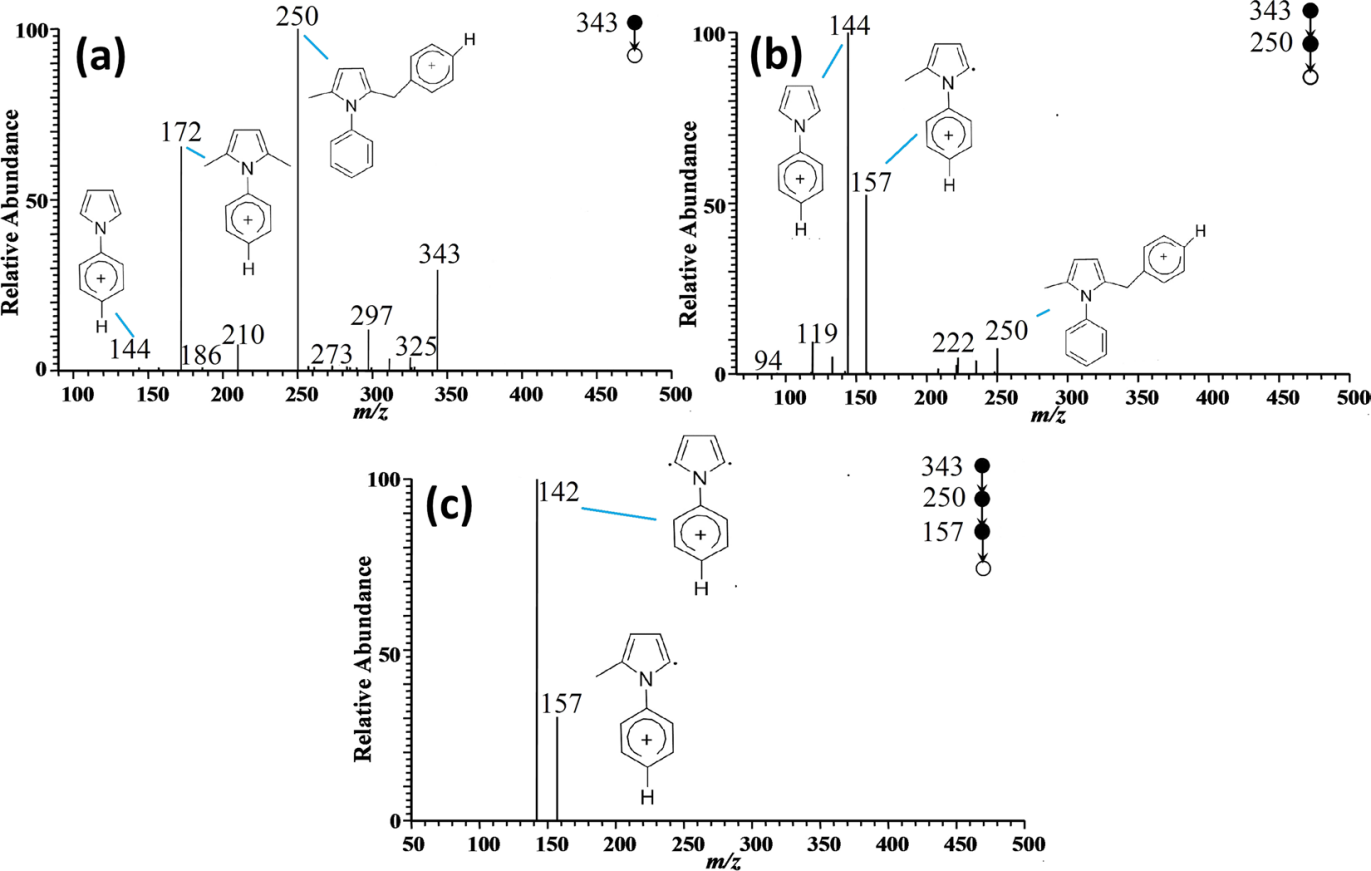
Tandem MS spectrum of m/z 343 and its possible fragmentation pathway. (**a**) MS^2^ spectrum of m/z 343; (**b**) MS^3^ spectrum of m/z 250; (**c**) MS^4^ spectrum of m/z 157.

By comparing the spectra of reactants (Fig. 4) and the spectra captured during the reaction (Fig. 6), it can be seen that the following ion peaks were produced during reaction: m/z 208, m/z 190, m/z 172 and m/z 343. Based on the reaction equation, the ion at m/z 172 was speculated to be phenyl-2,5-dimethyl pyrrole and the ion at m/z 343 to be its dimer. In order to determine the structure of these species, multi-stage CID analysis was carried out (see Figs. 7 and 8).

As is shown in Fig. 7, under CID mode, the peak at m/z 172 generated fragment peaks at m/z 157 and m/z 94. Based on the structure of phenyl-2,5-dimethyl pyrrole, it could be inferred that the methyl in the allylic position was unstable, and thus it might be eliminated under high collision energy. That is, the ion at m/z 172 lost mass of 15 to obtain a relatively stable structure of phenyl-2-methyl pyrrole radical cations at m/z 157. Meanwhile, it might be partly fractured, leading to generation of fragments at m/z 94 (aniline positive ion), m/z 131 (losing a hydrogen and combing one H_2_O) and m/z 130 (losing two hydrogen and combing one H_2_O), respectively. Therefore, the ion at m/z 172 was confirmed to be the target product of phenyl-2,5-dimethyl pyrrole.

The peaks at m/z 208 and m/z 190 were also studied in detail, both of which were produced successively as shown in Fig. 6. In the MS^2^ spectrum of m/z 208, three fragment ion peaks at m/z 190, m/z 94 and m/z 115 were observed (Fig. 7). Since m/z 94 and m/z 115 were ascribed to reactants of aniline positive ions and acetonylacetone positive ions, m/z 208 was speculated to be one of the intermediates. The generation of peak at m/z 190 could be due to m/z 208 losing a water molecule. Therefore, the structure of ion m/z 208 was assigned to phenyl-2,5-dyhydroxy-2,5-dimethyl pyrrole. MS^2^ of m/z 190 precursor ion produced the major fragment ion at m/z 172. The MS^3^ spectrum of m/z 172 product ion generated major ions of m/z 157, m/z 142, m/z 130 and m/z 94. These peaks were identical to the fragment peaks in MS^2^ spectrum of m/z 172. Therefore, the major fragment peak at m/z 172 resulting from m/z 190 was confirmed to be the target product of phenyl-2,5-dimethyl pyrrole. The ion at m/z 190 therefore was the intermediate. On the other hand, because m/z 208 could generate m/z 190 (phenyl-2-hydroxy-2,5-dimethyl pyrrole) by losing one water molecular, the peak at m/z 208 again was identified as an intermediate rather than byproduct.

CID analysis was also conducted for the ion peak at m/z 343 to determine its structure, which was previously presumed as the dimers of ion m/z 172. It can be seen from Fig. 8 that major fragment peaks at m/z 343, m/z 250 and m/z 172 could be detected from the MS^2^ spectra of m/z 343. Thus, it could be concluded that m/z 343 consisted of target products (m/z 172). Choosing m/z 250 as precursor, MS^3^ of m/z 250 showed that its fragment peaks were m/z 157 and m/z 144 respectively. Based on the analysis above, ion m/z 157 was confirmed to be phenyl-2-methyl pyrrole radical cations. On the other hand, given that the mass difference between m/z 250 and m/z 157 was 77 which was exactly the mass of benzene ring, the peak at m/z 250 was assigned as phenyl-2-methyl-5-phenyl pyrrole. As for the peak at m/z 157, further CID analysis was conducted. The MS^4^ of m/z 157 demonstrated that its major featured peak was m/z 142. That is, m/z 157 lost a methyl (mass of 15) from its side chain to obtain the fragment ion of m/z 142. As a result, m/z 343 was confirmed to be the protonated dimer of the target product.

### Reaction mechanism between aniline and acetonylacetone

**Figure 9:**
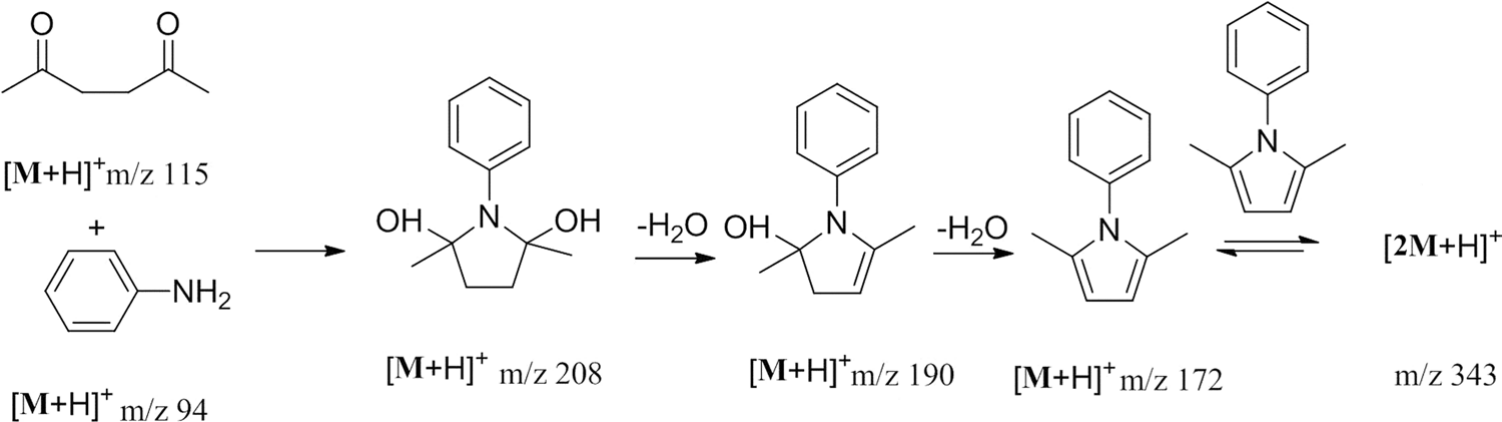
The mechanism of acetic acid-catalyzed reaction between aniline and acetonylacetone.

Based on the analysis above, the whole reaction process could be described as follows: being catalyzed by acetic acid, a lone pair of C=O in 2,5-hexanedione forms a coordinate covalent bond with a proton. Then, the resulting carbon becomes more susceptible to nucleophilic attack, and therefore nucleophilic addition reaction between aniline and acetonylacetone occurs. The reaction generated the ion of phenyl-2,5-dyhydroxy-2,5-dimethyl pyrrole (m/z 208). Subsequently, one water molecule was removed due to the β-elimination reaction, leading to the generation of phenyl-2,5-dimethyl pyrrole (m/z 172). On the other hand, further reaction between phenyl-2,5-dimethyl pyrrole resulted in formation of its dimer (m/z 343). The overall reaction mechanism was shown in Fig. 9. Noteworthy, from Figs. 3(a) and 6, it can be seen that m/z 343 disappeared eventually, indicating possible decomposition into target product.

## Conclusion

The pathway and mechanism of acetic acid-catalyzed reaction between aniline and acetonylacetone were studied using EESI-MS. Compared with conventional analytical methods, such as (HPLC-)MS or GC-MS, the use of EESI-MS allowed real-time monitoring of reactants, intermediates and other byproducts during the entire reaction. By studying the MS spectra, the structure of each component was confirmed and the reaction mechanism was deduced. The results showed that hydrogen proton of acetic acid enhanced the positive electrophilicity of carbon in acetonylacetone, which facilitated its nucleophilic reaction with aniline. As a result, water loss occurred and target product was produced. EESI-MS was demonstrated as an effective technique for the on-line direct analysis of reaction mechanism.

## Supplementary information

Supporting information
