# Supplementary material for: Real-time monitoring of the reaction between aniline and acetonylacetone using extractive electorspray ionization tandem mass spectrometry
Source: Sci Rep. 2019 Dec 17;9:19279. doi: 10.1038/s41598-019-55921-1 (PMC6917761; doi:10.1038/s41598-019-55921-1)
Supplement: Supplementary file 1 — Supporting information [file 41598_2019_55921_MOESM1_ESM.docx]

*Supporting information*

**Real-time monitoring of the reaction between aniline and acetonylacetone using extractive electorspray ionization tandem mass spectrometry**

Xinglei Zhang^1^, Miaorong Pei^1^, Debo Wu^*1^, Shuiping Yang^1^ and Zhanggao Le^1^

*^1^Jiangxi Key Laboratory for Mass Spectrometry and Instrumentation, East China University of Technology, Nanchang 330013 People’s Republic of China*

*Corresponding Author:

Dr. Debo Wu

Jiangxi Key Laboratory for Mass Spectrometry and Instrumentation,

East China University of Technology,

Nanchang 330013

People’s Republic of China

Email:  wudebo@hotmail.com

Phone:  +86-0791-83896370. Fax: +86-0791-83896370.

**Fig S1**


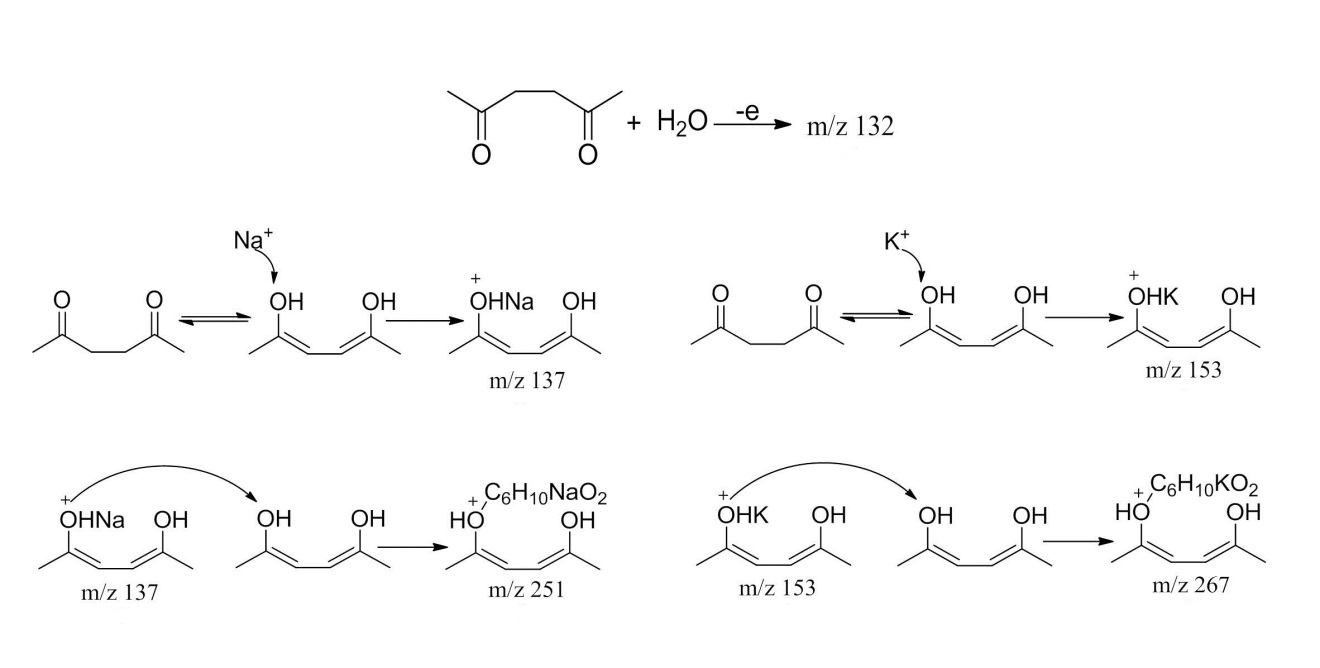


**Figure S1** Illustration of possible reaction between acetonylacetone and Na^+^, K ^+^ ions

**Fig S2**


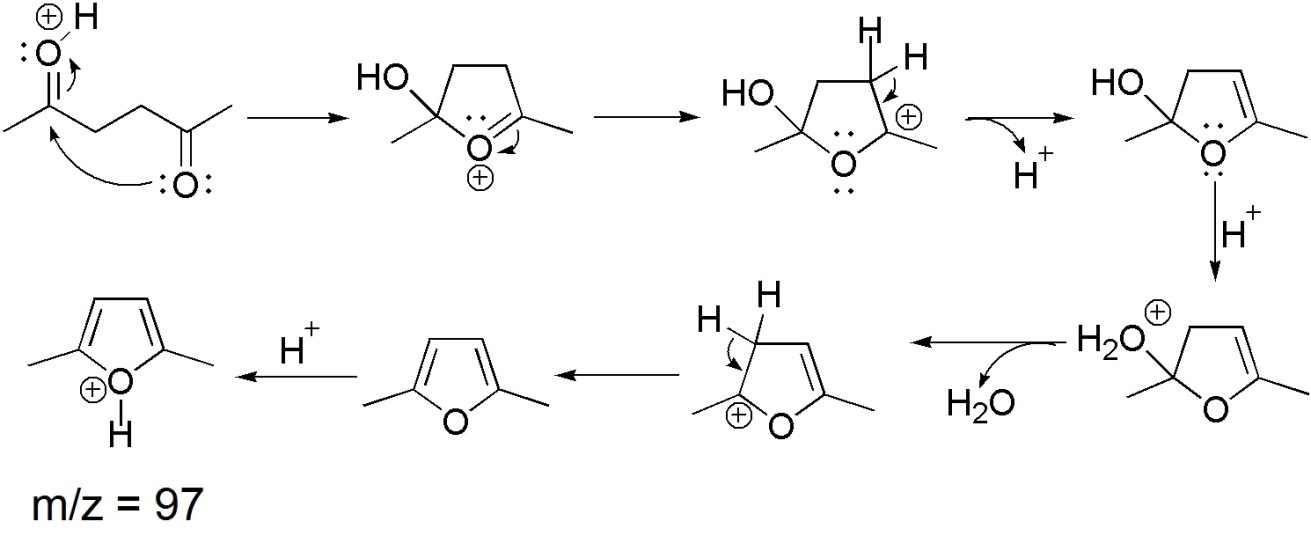


**Figure S2** Proposed mechanism for the formation of m/z 97 (protonated 2,5-dimethylfuran).

**Fig S3**


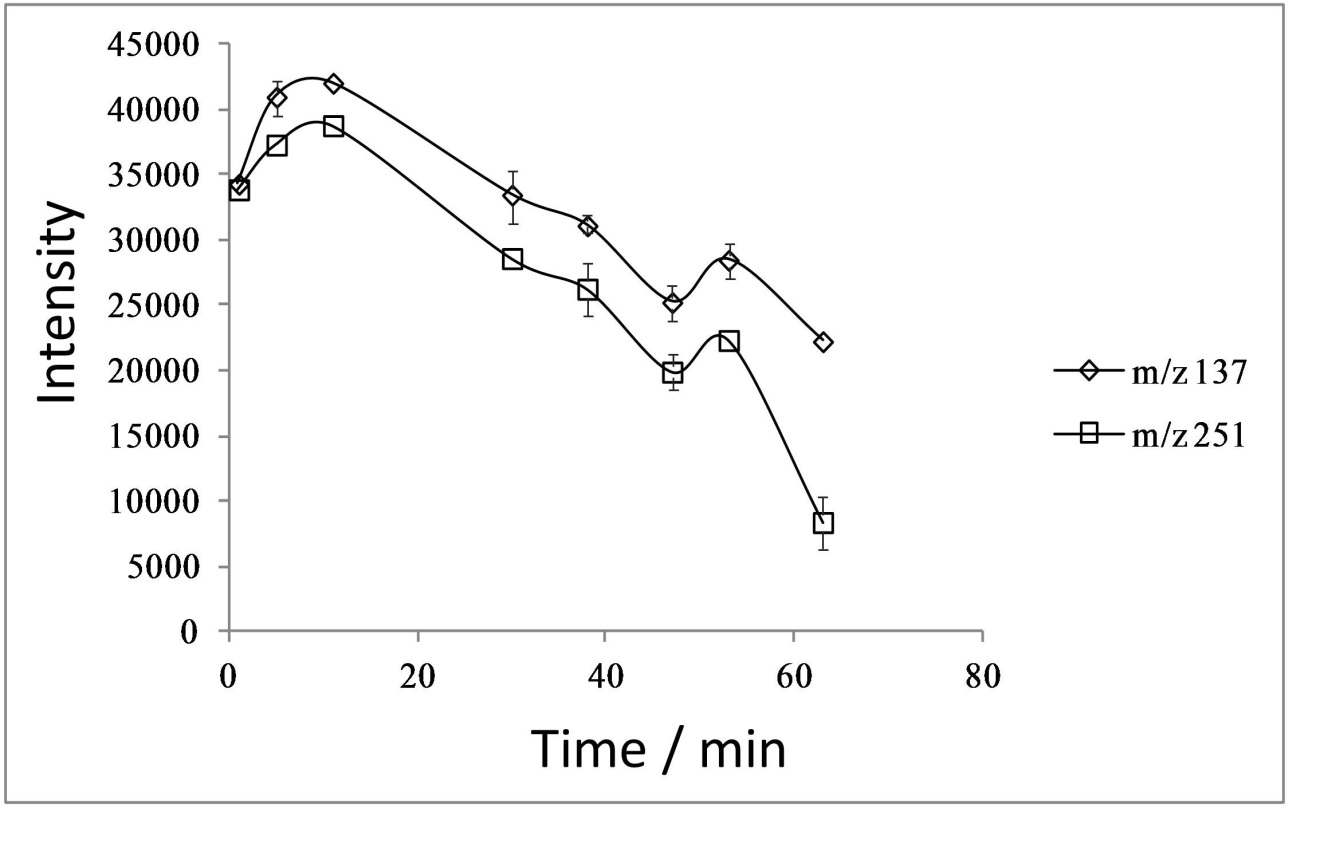


**Figure S3** The intensity behavior of m/z 137 and m/z 251with the increase of reaction time.
